# Parsing reward sensitivity reveals distinct relationships with energy intake, metabolic markers, physical activity and fitness

**DOI:** 10.1017/neu.2024.63

**Published:** 2025-01-30

**Authors:** Evelyn Kiive, Urmeli Katus, Diva Eensoo, Inga Villa, Jarek Mäestu, Toomas Veidebaum, Jaanus Harro

**Affiliations:** 1 Division of Special Education, Department of Education, University of Tartu, Tartu, Estonia; 2 Department of Family Medicine and Public Health, University of Tartu, Estonia, Tartu, Tartumaa; 3 Department of Chronic Diseases, National Institute for Health Development, Tallinn, Harjumaa, Estonia; 4 Division of Exercise Biology, Institute of Sport Sciences and Physiotherapy, University of Tartu, Tartu, Tartumaa, Estonia; 5 Division of Neuropsychopharmacology, Department of Chemistry, University of Tartu, Tartu, Tartumaa, Estonia

**Keywords:** reward sensitivity, metabolism, dietary intake, physical activity, cardiorespiratory fitness

## Abstract

Rewards are rewarding owing to their hedonic or metabolic value. Individual differences in sensitivity to rewards are predictive of mental health problems but may reflect variation in metabolic types. We have assessed the association of two distinguishable aspects of reward sensitivity, openness to rewards (the striving towards multiple rewards) and insatiability by reward (the strong pursuit and fixation to a particular reward), with measures of metabolism and activity in a longitudinal study of representative birth cohort samples. We used data of the Estonian Children Personality Behaviour and Health Study (original n = 1238) collected at age 15, 18 and 25. Reward sensitivity and physical activity were self-reported during a laboratory visit, when also blood sampling, measurement of blood pressure, height and weight, aerobic exercise testing and the diet interview, after the participants had kept food diary, took place. In the younger cohort, physical activity was also assessed by accelerometry at age 18 and 25. Across adolescence and young adulthood, openness to rewards was positively associated with physical activity and negatively with blood pressure and serum levels of glucose, insulin and cholesterol levels. In contrast, insatiability by reward was positively associated with serum triglyceride levels and negatively with energy intake and cardiorespiratory fitness. In conclusion, the two facets of reward sensitivity have a fairly different association with a variety of metabolic and health-related measures. This may explain the variable findings in literature, and suggests that individual differences in reward sensitivity are part of a complex physiological variability, including energy expenditure profiles.

## Graphical abstract

**Figure:**
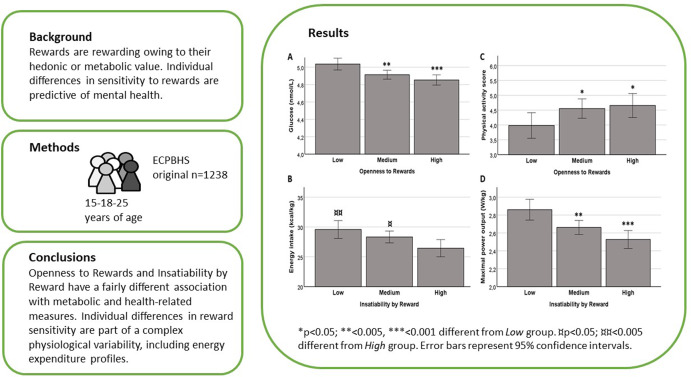

## Significant outcomes

- In birth cohort representative samples, sensitivity to rewards was associated with physical activity, aerobic fitness, energy intake and serum markers of metabolism.
- These associations were clearly different for the two distinguishable aspects of reward sensitivity: Openness to Rewards (striving towards multiple rewards) versus Insatiability by Reward (fixation to a specific reward).

## Limitations

- While the sample was highly representative and data from three observations over 10 years were used, the study represents one setting and hence requires replication.
- This study was observational, and causality cannot be implied in either direction. Studies on underlying biological mechanisms are needed to guide the design of experimental models.

## Introduction

Sensitivity to rewards refers to the degree to which an individual’s behaviour is motivated by reward-relevant stimuli (Gray, 1970; Carver and White, 1994; Corr and Cooper, 2016). Indeed, individuals vary considerably in their sensitivity to rewards (Carver and White, 1994; Kim *et al*., 2015; Corr, 2016), and this has major implications for the development of multiple psychiatric disorders (Holroyd and Umemoto, 2016). On the other hand, reward sensitivity appears to be conserved across evolution, given that many mammalian species show reward-related behavioural patterns similar to humans (Spear, 2011) and share its neurobiological substrate (Panksepp, 1998). From an evolutionary perspective, reward sensitivity is an absolutely vital construct; all necessities for survival and procreation are considered naturally rewarding, and rewards also reinforce the associated behaviours (Blaukopf and DiGirolamo, 2007; Sullivan *et al*., 2008). Reward sensitivity is a trait strongly dependent on the activity of the mesolimbic dopamine system, which attributes salience to stimuli and makes them an incentive (Robinson and Berridge, 1993) and supports seeking out resources (Panksepp, 1998) by increasing appetitive behavioural arousal and facilitating sensitivity to rewards while promoting adherence to safety (Ikemoto and Panksepp, 1999). Dopamine neurones fire in correspondence with the subjective value of reward (Hill *et al*., 2024) and can regulate dietary choices (Wu *et al*., 2024), promote habitual physical activity (Ruiz-Tejada *et al*., 2022) and balance feeding and physical exercise-related energy expenditure (Walle *et al*., 2024). Indeed, animals evolved as reward seekers, but reward-seeking behaviour entails severe costs and benefits (Murray *et al*., 2011). A life of movement necessitates decisions about where to move, when and whether to do so. Animals must decide whether to abandon the relative safety that often comes from staying still to forage for nutrients and other rewards. In turn, dietary sugar and lipids can act as functional modulators of the dopaminergic reward circuit (May *et al*., 2020; Berland *et al*., 2021). The physiological checks and balances have evolved over millions of years in an effortful search for safety, energy and nutrients, in stark contrast with the modern lifestyle of humans, which is now superimposed over the drives shaped by evolution, with obvious consequences to public health (Higgs *et al.*, 2017; Wiss *et al*., 2018; Woessner *et al*., 2021).

Different strategies of exploration of the physical environment and seeking out rewards have been explained as an outcome of the interaction between individual traits of anxiety and motivation (Harro, 2010), with reward sensitivity being embedded in the latter. However, individual differences in energy requirement may also play a role. These may also explain the variable level of health risks brought about by sedentary behaviour and excessive energy intake (Wells, 2017; Reddon *et al*., 2018). The variability in energy requirement is affected by numerous physiological factors (National Academies of Sciences *et al*., 2023). Besides resting energy expenditure as the most significant component of total energy expenditure, physical activity is the second largest component. Metabolic responses to food, age, sex, body size, body composition and climate also contribute.

Some evidence does suggest that personality and metabolism are related. For example, individuals who scored lower on neuroticism and higher on extraversion, openness and conscientiousness had significantly higher energy expenditure at peak walking pace, suggesting an association of psychological processes with energy homeostasis and individual differences in aerobic capacity (Terracciano *et al.,* 2013). Blum *et al*. (1996) have described the *reward deficiency syndrome,* proposing that individuals who have functional genetic deviations of one or more of the components of the reward pathway tend to be less satisfied with natural rewards and seek enhanced stimulation of these pathways, for example, through drugs, food or engaging in dangerous sports. The syndrome is associated with hypo-dopaminergic function resulting in abnormal craving behaviour, and alterations in dopamine reward circuits can lead to pathologic food consumption, increased fat synthesis and digestive disorders (Blum *et al.*, 2014). Miró-Padilla *et al*. (2023) studied the impact of personality and the volume of reward-related brain areas on individual differences in voluntary physical activity, objectively measured by an accelerometer, in young adults. Smaller volumes of the right anterior cingulate cortex were related to lower scores in reward sensitivity, which contributed to explaining low levels of daily physical activity. The authors suggested that individual differences in the activity of the reward system constrain a behavioural repertoire characterised by more vigorous and frequent actions aimed at obtaining rewards, with practising physical activity being a good example of this behaviour (Miró-Padilla *et al*., 2023).

Intuitively, reward sensitivity should be in positive association with the consumption of palatable foods. Indeed, a positive association between sensitivity to reward and unhealthy snacks and sugar-sweetened beverage consumption in adolescents has been reported (De Cock *et al.*, 2016). In addition to tastiness, foods are also rewarding because of their caloric value: van Rijn *et al*. (2016) found that neural responses to oral calories from a maltodextrin solution are modulated by reward sensitivity in reward-related areas such as the caudate nucleus, amygdala and anterior cingulate cortex. However, a systematic review concluded that while reward sensitivity, primarily measured by the Sensitivity to Punishment and Sensitivity to Reward Questionnaire (SPSRQ) or Behavioural Inhibition/Activation System Scale (BIS/BAS), is positively associated with binge eating and emotional, external, hedonic and excessive eating behaviours as well as obesity-related outcomes, the effect sizes are small to moderate (Sutton *et al*., 2022). Indeed, there is also a host of research not finding any association of reward sensitivity with food intake or body composition (e.g. Scholten *et al*., 2014; Vandeweghe *et al*., 2017; Goldschmidt *et al*., 2019; Jonker *et al*., 2019).

Individual energy expenditure and intake differences depend on the underlying metabolic phenotype, such as thrifty versus spendthrift (e.g. Piaggi *et al*., 2018; Hollstein *et al*., 2020). A thrifty phenotype is characterised by a greater decrease in energy expenditure with fasting associated with its smaller increase in response to overfeeding. A thrifty phenotype is thus prone to less weight loss during underfeeding but greater weight gain with overfeeding. This phenotype is considered metabolically efficient and presumably engenders a risk of obesity. By contrast, a spendthrift phenotype markedly increases energy expenditure in response to overfeeding but shows only small decreases with underfeeding. Individuals with different energy profiles may differ in their reward processing, as the homeostatic and hedonic systems interact to regulate body weight (Berthoud *et al*., 2017). The functional interaction of the hypothalamus with the corticolimbic system and the brainstem provides the emotional, cognitive and executive support to the drive to consume food.

The relatively inconsistent association of reward sensitivity with dietary intake and body composition may however derive from the heterogeneity of the reward sensitivity construct. We have recently shown in a birth cohort representative sample that reward sensitivity can be parsed into two independent components: one that represents striving towards multiple rewards (Openness to Rewards) and the other characterising the strong pursuit and fixation to a particular reward (Insatiability by Reward) (Pulver *et al*., 2020a). Compared to existing reward sensitivity models, for example, with the BIS/BAS framework, the Reward Openness and Insatiability Scale (ROIS) does not, unlike the behavioural activation system, assess the affective arousal or drive intensity associated with the obtaining or anticipating of rewards. Instead, it seeks to distinguish an individual’s tendency to remain receptive to a broad array of, particularly novel, rewards from a tendency to fixate on a specific reward due to impaired impulse control.

These two components of reward sensitivity, Openness to Rewards and Insatiability by Reward, were distinctly related to the affective neuroscience personality traits, and only the latter was associated with symptoms of attention deficit hyperactivity disorder (ADHD). Only Insatiability by Reward was associated with body weight, body mass index (BMI), sum of five skinfolds, waist circumference, hip circumference and waist-to-height ratio in adolescents and young adults (Katus *et al*., 2020a). Another interesting finding was the complex relationship of the two components of reward sensitivity with aggressiveness (Pulver *et al*., 2020b). Thus, while in the whole sample, aggressiveness was in positive association with Insatiability by Reward, there was a distinction in one fifth of the sample, the homozygotes for an aggressiveness-related minor variant of the orexin/hypocretin receptor type 1 encoding gene *HCRTR1* (Harro *et al*., 2019): in *HCRTR1* rs2271933 A/A homozygotes, aggressiveness was, exceptionally, related to Openness to Rewards. Given the central role of orexins in reward processing and motivation (Mahler *et al*., 2014; Baimel *et al*., 2015), glucose homeostasis (Tsuneki *et al*., 2010), promotion of foraging and homeostatic eating (Barson, 2020), but also in cardiorespiratory system (Shahid *et al*., 2012) and voluntary physical exercise (Tesmer *et al*., 2024), and considering the apparently dual association of orexin receptor genotype in shaping reward sensitivity (see Pulver *et al*., 2020b), we aimed at assessing the association of the two distinct aspects of reward sensitivity with dietary intake, metabolic markers, physical activity and cardiorespiratory fitness in this longitudinally studied birth cohort representative sample.

## Materials and methods

### Study sample

This study was carried out using a sample from the Estonian Children’s Personality Behaviour and Health Study (ECPBHS). The rationale of the formation of the original sample and the study procedure have been described previously (Harro *et al*., 2001). Of the eligible group, 79.1% participated, and the original sample consisted of 583 subjects aged 9 years and 593 subjects aged 15 years. Follow-up studies took place at ages 15, 18 and 25 years. Written informed consent was obtained from the participants and, in the case of minors, also from their parents. The study was approved by the Ethics Review Committee on Human Research of the University of Tartu and conducted in accordance with the Declaration of Helsinki.

### Measurement of reward sensitivity

Reward sensitivity was assessed using the ROIS; the construction and description of ROIS have been described in detail elsewhere (Pulver *et al*., 2020a). ROIS comprises 28 items, divided equally between two higher-order factors: *Openness to Rewards*, a strive towards a multiplicity of rewards, and *Insatiability by Reward*, characterised by an excessive fixation on a particular reward. *Openness to Rewards* includes *Excitement and Novelty*, with items reflecting the search for new experiences and excitement, and *Social Experiences*, with items primarily associated with sociability and social exchange. *Insatiability by Reward* comprises *Excessive Spending*, with items related to impulsive buying and excessive spending, and *Giving in to Cravings*, with items related to low self-control and trouble in resisting temptations. Personality information for analysis by ROIS was collected at age 25 or 33. The mean item score was used in statistical analysis.

### Dietary intake

In different study waves, dietary food intake recall was used for 24 h, 48 h or 72 h (Joost *et al*., 2019). Participants were asked to complete a diet record at home during the day(s) before the study day. A face-to-face interview was performed on the study day. Data on portion size that was not recorded in the food diary were estimated using pictures of portion sizes (Haapa *et al*., 1985). Where data from 2 or 3 days were available, the mean nutrient intake relative energy intake per day was calculated. Dietary intake was assessed using the Finnish Micro-Nutrica Nutritional Analysis programme adapted to include Estonian foods, Estonian version 2.0 (Tallinn University of Technology, Food Processing Institute, Estonia) and using the NutriData food consumption database, versions 4.0–7.0 (National Institute for Health Development, Estonia).

### Cardiorespiratory fitness and physical activity

Cardiorespiratory fitness was determined using a cycle-ergometer (Tunturi T8, Tunturi New Fitness B.V., Finland) test with progressively increasing workload until exhaustion and was defined as maximal power output calculated per kilogram of body weight (W/kg). The procedure has been described in detail elsewhere (Lätt *et al*., 2018). Physical activity was assessed using self- and parent-reported questionnaires. Individual physical activity scores were calculated as previously described (Katus *et al*., 2020b), and standardised (z-scores) scores of each year were used. In the younger cohort at ages 18 and 25, physical activity was also measured by uniaxial accelerometer GT1M (ActiGraph, Monrovia, CA, USA) to detect vertical accelerations ranging in magnitude from 0.05 to 2.00 g with a frequency response of 0.25–2.50 Hz. Each participant was asked to carry the monitor on the right hip for seven consecutive days during the awakening hours, and steps per day were used in the analysis.

### Blood pressure, metabolic markers and blood lipid measurements

Resting systolic blood pressure (SBP) and diastolic blood pressure (DBP) were measured in a laboratory setting on the left arm with an automatic oscillometric method from the left arm in a sitting position. Five consecutive measurements were made at 2-min intervals, and the mean value was used in the analysis.

Venous blood samples were taken from the antecubital vein after a fast of 8–12 h and analysed in a certified clinical laboratory. Insulin resistance was estimated using the homeostatic model assessment (HOMA) index, which was calculated as fasting glucose (mmol/l) × fasting insulin (mU/l)/22.5 (Matthews *et al*., 1985). Fasting basal cholesterol, high-density lipoprotein (HDL) cholesterol and low-density lipoprotein (LDL) cholesterol, as well as triglyceride (TRG) levels, were measured by conventional techniques in the Central Laboratory of the Tartu University Hospital and presented in mmol/l (Tomson *et al*., 2011).

### Statistical analysis

Longitudinal associations between reward sensitivity and dietary intake, cardiorespiratory fitness, blood pressure and metabolic measures were assessed using the linear mixed-effects regression models with random intercept and random slope. Mixed models are well suited for longitudinal data as they account for the correlations between repeated measurements within each subject, and in such data, the variance is often heterogeneous over time. These models allow different correlation matrices as observations may be correlated in several different ways. In addition, unlike repeated measures ANOVA, mixed models include all available data to the analysis (Detry and Ma, 2016). Mixed models accommodate unbalanced data patterns using all available observations in the analysis assuming that missingness is random, that is, independent of unobserved measurements but dependent on the observed measurements. Imputation techniques were not applied. Dependent measures were dietary intake, cardiorespiratory fitness, blood pressure and metabolic measures at baseline (age 15 years) and follow-up points (18 years and 25 years). The reward sensitivity score was defined as the independent variable. Time was treated as a continuous variable. The likelihood ratio test was used to assess the goodness of fit of the statistical models. Unstructured or exchangeable covariance structure and restricted maximum likelihood method were used. In the younger cohort, accelerometry data were available but only for two timepoints (18 and 25 years), and thus less complex mixed-effects regression models were fitted with only random intercept and using compound symmetric covariance structure. For illustrative purposes, the relationship of aspects of reward sensitivity with selected measures at age 25 was also compared by reward sensitivity groups (lowest and highest quartile and medium 50%) after one-way ANOVA analysis and subsequent *post hoc* LSD tests.

## Results

**Figure 1.**
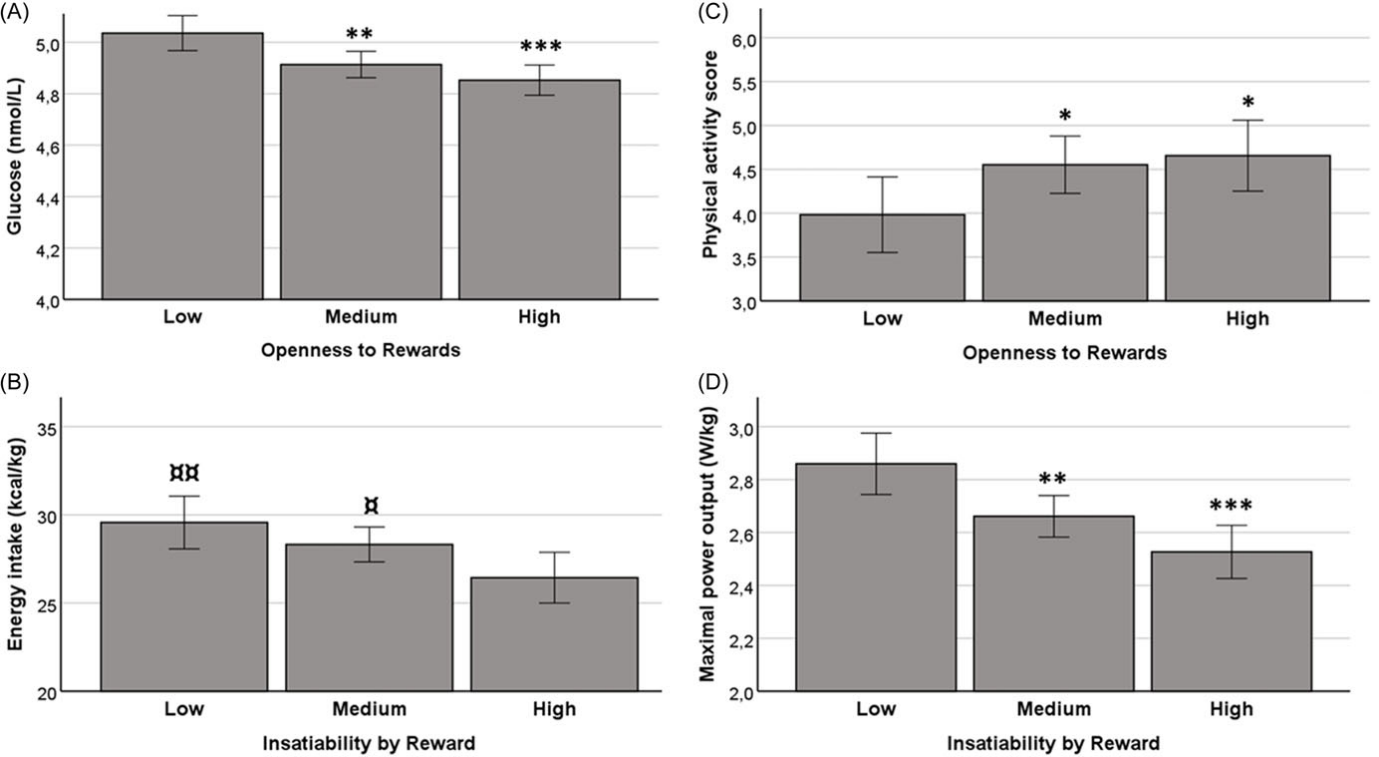
Aspects of reward sensitivity and selected measures of metabolism and lifestyle at age 25. This illustrative analysis is based on quartile groups of rewards sensitivity facets. Blood glucose level (A), energy intake (B), physical activity score (C) and cardiovascular fitness by maximal power output (D) at age 25 years in ECPBHS participants with low (25%), medium (50%) or high (25%) reward sensitivity. *p < 0.05; **<0.005, ***<0.001 different from *Low* group. ^¤^ *p* < 0.05; ^¤¤^ *p* < 0.005 different from *High* group. Error bars represent 95% confidence intervals.

Results from the linear mixed-effects regression models showing the relationship between reward sensitivity scores and glucose and lipid metabolism from 15 to 25 years of age are presented in Table 1. Blood glucose, insulin and HOMA index were inversely associated with Openness to Rewards (OR) and especially with higher Social Experience subscale. The relationship of Openness to Rewards with glucose levels at age 25 is depicted in Figure 1A (F2,741 = 7.81; *p* < 0.001; *η* = 0.021). A trend of negative association of Openness to Rewards with total cholesterol levels was also noted. Furthermore, a negative association between OR Social Experience subscale (*p* < 0.05) and LDL cholesterol was observed, while no associations between reward sensitivity and HDL cholesterol were detected (Table 1).

**Table 1.** Estimated main effects (mean and 95% CI) of the ECPBHS sample in insulin resistance and food lipid concentration (mmol/l), from 15 to 25 years of age by reward sensitivity score according to the linear mixed-effects regression model

	Coefficient	95% CI	*p* value		Coefficient	95% CI	*p* value
**Glucose (mmol/l)**				**Glucose (mmol/l)**			
Openness to Rewards (OR)	−0.057	−0.101; –0.013	**0.011**	Insatiability by Reward (IR)	−0.016	−0.056; 0.024	0.438
OR Excitement and Novelty	−0.002	−0.041; 0.036	0.902	IR Excessive Spending	−0.010	−0.042; 0.021	0.528
OR Social Experience	−0.075	−0.111; –0.039	**<0.001**	IR Giving in to Cravings	−0.016	−0.053; 0.020	0.374
**Insulin (mU/l)**				**Insulin (mU/l)**			
Openness to Rewards (OR)	−0.578	−1.072; –0.084	**0.022**	Insatiability by Reward (IR)	0.237	−0.211; 0.685	0.300
OR Excitement and Novelty	−0.342	−0.779; 0.094	0.124	IR Excessive Spending	0.312	−0.042; 0.666	0.084
OR Social Experience	−0.485	−0.892; –0.078	**0.020**	IR Giving in to Cravings	−0.071	−0.481; 0.338	0.733
**HOMA (units)**				**HOMA (units)**			
Openness to Rewards (OR)	−0.156	−0.278; –0.034	**0.012**	Insatiability by Reward (IR)	0.044	−0.067; 0.054	0.440
OR Excitement and Novelty	−0.073	−0.180; 0.035	0.187	IR Excessive Spending	0.066	−0.022; 0.153	0.141
OR Social Experience	−0.148	−0.249; –0.048	**0.004**	IR Giving in to Cravings	−0.033	−0.135; 0.068	0.518
**CHL (mmol/l)**				**CHL (mmol/l)**			
Openness to Rewards (OR)	−0.081	−0.163; –0.003	0.050	Insatiability by Reward (IR)	0.034	−0.038; 0.105	0.356
OR Excitement and Novelty	−0.063	−0.134; 0.009	0.085	IR Excessive Spending	0.013	−0.044; 0.070	0.650
OR Social Experience	−0.055	−0.123; 0.012	0.107	IR Giving in to Cravings	0.042	−0.026; 0.109	0.226
**HDL-C (mmol/l)**				**HDL-C (mmol/l)**			
Openness to Rewards (OR)	0.032	−0.005; 0.069	0.090	Insatiability by Reward (IR)	0.004	−0.028; 0.037	0.789
OR Excitement and Novelty	0.027	−0.005; 0.059	0.103	IR Excessive Spending	−0.004	−0.030; 0.022	0.760
OR Social Experience	0.019	−0.011; 0.050	0.213	IR Giving in to Cravings	0.013	−0.017; 0.044	0.390
**LDL-C (mmol/l)**				**LDL-C (mmol/l)**			
Openness to Rewards (OR)	−0.070	−0.147; 0.006	0.072	Insatiability by Reward (IR)	0.032	−0.035; 0.098	0.350
OR Excitement and Novelty	−0.036	−0.103; 0.031	0.294	IR Excessive Spending	0.012	−0.041; 0.065	0.661
OR Social Experience	−0.064	−0.127; –0.001	**0.047**	IR Giving in to Cravings	0.040	−0.023; 0.103	0.210
**TRG (mmol/l)**				**TRG (mmol/l)**			
Openness to Rewards (OR)	−0.014	−0.055; 0.027	0.499	Insatiability by Reward (IR)	0.057	0.021; 0.092	**0.002**
OR Excitement and Novelty	−0.023	−0.059; 0.012	0.199	IR Excessive Spending	0.050	0.022; 0.078	**<0.001**
OR Social Experience	0.002	−0.031; 0.036	0.911	IR Giving in to Cravings	0.030	−0.004; 0.063	0.082

Similar associations with glucose and lipid metabolism were not present with the other facet of reward sensitivity, Insatiability by Reward. Instead, linear mixed-effects regression models suggested a significant negative relationship between Insatiability by Reward and its both subscales and body weight-adjusted daily energy intake, protein, lipid and carbohydrate intake (Table 2; Figure 1B for energy intake; F2,725 = 4.73; *p* < 0.01; *η*=0.013). In contrast, a subscale of Openness to Rewards, Excitement and Novelty, was positively associated with protein intake as a percentage from daily energy intake (Table 2).

**Table 2.** Estimated main effects (mean and 95% CI) of the ECPBHS sample in daily energy intake (kcal), nutrient intake (g/kg) and nutrient intake as a percentage from daily energy intake (E%) from 15 to 25 years of age by reward sensitivity score according to the linear mixed-effects regression model

	Coefficient	95% CI	*p* value		Coefficient	95% CI	*p* value
**Energy intake (kcal/kg)**				**Energy intake (kcal/kg)**			
Openness to Rewards (OR)	0.021	−1.116; 1.157	0.972	Insatiability by Reward (IR)	−1.667	−2.651; –0.683	**0.001**
OR Excitement and Novelty	0.488	−0.511; 1.488	0.338	IR Excessive Spending	−1.347	−2.107; –0.587	**0.001**
OR Social Experience	−0.409	−1.347; 0.530	0.393	IR Giving in to Cravings	−1.134	−2.071; –0.197	**0.018**
**Protein (g/kg)**				**Protein (g/kg)**			
Openness to Rewards (OR)	0.016	−0.026; 0.058	0.460	Insatiability by Reward (IR)	−0.074	−0.112; –0.036	**<0.001**
OR Excitement and Novelty	0.035	−0.001; 0.072	0.060	IR Excessive Spending	−0.052	−0.082; –0.022	**0.001**
OR Social Experience	−0.010	−0.044; 0.025	0.585	IR Giving in to Cravings	−0.065	−0.099; –0.030	**<0.001**
**Lipids (g/kg)**				**Lipids (g/kg)**			
Openness to Rewards (OR)	0	−0.052; 0.052	0.993	Insatiability by Reward (IR)	−0.058	−0.104; –0.013	**0.012**
OR Excitement and Novelty	0	−0.045; 0.045	0.992	IR Excessive Spending	−0.046	−0.081; –0.011	**0.010**
OR Social Experience	−0.017	−0.059; 0.025	0.429	IR Giving in to Cravings	−0.045	−0.087; –0.003	**0.035**
**Carbohydrates (g/kg)**				**Carbohydrates (g/kg)**			
Openness to Rewards (OR)	−0.020	−0.160; 0.119	0.776	Insatiability by Reward (IR)	−0.247	−0.367; –0.126	**<0.001**
OR Excitement and Novelty	0.009	−0.117; 0.134	0.889	IR Excessive Spending	−0.156	−0.253; –0.058	**0.002**
OR Social Experience	−0.061	−0.176; 0.054	0.301	IR Giving in to Cravings	−0.138	−0.255; –0.020	**0.021**
**Protein E%**				**Protein E%**			
Openness to Rewards (OR)	0.260	−0.015; 0.535	0.064	Insatiability by Reward (IR)	−0.010	−0.237; 0.217	0.932
OR Excitement and Novelty	0.270	0.028; 0.512	**0.029**	IR Excessive Spending	0.006	−0.173; 0.186	0.945
OR Social Experience	0.119	−0.108; 0.347	0.304	IR Giving in to Cravings	−0.027	−0.254; 0.200	0.818
**Lipids E%**				**Lipids E%**			
Openness to Rewards OR)	−0.067	−0.636; 0.503	0.819	Insatiability by Reward (IR)	0.158	0.340; 0.656	0.533
OR Excitement and Novelty	−0.060	−0.562; 0.442	0.814	IR Excessive Spending	0.074	−0.320; 0.468	0.714
OR Social Experience	−0.035	−0.505; 0.435	0.885	IR Giving in to Cravings	0.179	−0.292; 0.651	0.456
**Carbohydrates E%**				**Carbohydrates E%**			
Openness to Rewards (OR)	−0.444	−1.124; 0.237	0.201	Insatiability by Reward (IR)	−0.352	−0.963; 0.258	0.258
OR Excitement and Novelty	−0.314	−0.914; 0.286	0.304	IR Excessive Spending	−0.249	−0.732; 0.235	0.313
OR Social Experience	−0.335	−0.897; 0.228	0.244	IR Giving in to Cravings	−0.279	−0.858; 0.299	0.344

The analysis also revealed a significant negative relationship between Openness to Rewards and SBP and DBP. Furthermore, a positive association between Openness to Rewards and its subscales and physical activity was noted (Table 3 and Figure 1C; F2,761 = 2.98; *p* < 0.05; *η* = 0.01). Again, such associations were not found for Insatiability by Reward, but this aspect of reward sensitivity was negatively associated with cardiorespiratory fitness (Table 3; Figure 1D; F2,711 = 9.14; *p* < 0.001; *η* = 0.025). In contrast, maximal power output was in positive association with the Excitement and Novelty subscale of Openness to Rewards.

**Table 3.** Estimated main effects (mean and 95% CI) of the ECPBHS sample in blood pressure, cardiovascular fitness expressed as maximum power output (MPO; W/kg) and physical activity from 15 to 25 years of age by reward sensitivity score according to the linear mixed-effects regression model

	Coefficient	95% CI	*p* value		Coefficient	95% CI	p value
**Systolic BP (mmHg)**				**Systolic BP (mmHg)**			
Openness to Rewards (OR)	−2.259	−3.500; –1.017	**<0.001**	Insatiability by Reward (IR)	0.464	−0.651; 1.578	0.415
OR Excitement and Novelty	−1.038	−2.132; 0.057	0.063	IR Excessive Spending	0.353	−0.531; 1.236	0.434
OR Social Experience	−2.162	−3.187; –1.138	**<0.001**	IR Giving in to Cravings	0.327	−0.696; 1.351	0.531
**Diastolic BP (mmHg)**				**Diastolic BP (mmHg)**			
Openness to Rewards (OR)	−1.341	−2.075; –0.608	**<0.001**	Insatiability by Reward (IR)	0.355	−0.277; 0.986	0.271
OR Excitement and Novelty	−0.999	−1.645; –0.353	**0.002**	IR Excessive Spending	0.333	−0.168; 0.833	0.192
OR Social Experience	−0.949	−1.556; –0.343	**0.002**	IR Giving in to Cravings	0.149	−0.456; 0.755	0.629
**Cardiovascular fitness (MPO, W/kg)**				**Cardiovascular fitness (MPO, W/kg)**			
Openness to Rewards (OR)	−0.008	−0.081; 0.065	0.830	Insatiability by Reward (IR)	−0.156	−0.224; –0.087	**<0.001**
OR Excitement and Novelty	0.083	0.013; 0.152	**0.020**	IR Excessive Spending	−0.093	−0.143; –0.043	**<0.001**
OR Social Experience	−0.044	−0.109; 0.022	0.193	IR Giving in to Cravings	−0.128	−0.187; –0.068	**<0.001**
**sPA score**				**sPA score**			
Openness to Rewards (OR)	0.248	0.168; 0.328	**<0.001**	Insatiability by Reward (IR)	−0.060	−0.132; 0.013	0.106
OR Excitement and Novelty	0.174	0.101; 0.246	**<0.001**	IR Excessive Spending	−0.032	−0.088; 0.024	0.262
OR Social Experience	0.158	0.090; 0.225	**<0.001**	IR Giving in to Cravings	−0.061	−0.128; 0.006	0.073
Accelerometry (steps/day)*				Accelerometry (steps/day)*			
Openness to Rewards (OR)	1127.7	246.5; 2008.9	**0.012**	Insatiability by Reward (IR)	–234.7	–909.0; 439.5	0.495
OR Excitement and Novelty	549.3	–237.7; 1336.3	0.171	IR Excessive Spending	–155.1	–688.4; 378.2	0.569
OR Social Experience	941.2	258.2; 1624.1	**0.007**	IR Giving in to Cravings	–206.5	–856.6; 443.7	0.534

sPA score, standardized physical activity score; *analysis is restricted to data of the younger cohort collected at ages 18 and 25.

## Discussion

Herewith, we describe how parsing reward sensitivity can help to reveal the association of this behavioural trait with metabolic markers, dietary intake, physical activity and cardiorespiratory fitness.

Openness to Rewards, an aspect of reward sensitivity corresponding to striving towards reward variety, was associated throughout adolescence and young adulthood with lower fasting glucose and insulin levels and HOMA index. This association was rather owing to one of the two subscales of Openness to Rewards, which specifically addresses rewarding aspects of social relationships. Social competence is negatively affected by impulsivity traits, and impulse control may be metabolically expensive process, creating higher glucose demand (Gailliot and Baumeister, 2007). Low blood glucose levels are indeed associated with impulsive aggression (DeWall *et al*., 2011); administration of glucose can reduce aggression in response to provocation among people high in trait aggression (Denson *et al*., 2010) and reduce disinhibition in a continuous performance test (Flint and Turek, 2003), whereas behavioural flexibility depends on glucoregulation (Riby *et al*., 2017).

Higher Openness to Rewards was also associated with higher habitual physical activity. This appears as fitting with the construct of an active, energetic reward seeker and may relate to the similar positive association with cardiovascular fitness and lower blood pressure and the trend of lower blood lipid levels. It should be noted that Openness to Rewards did not associate with body weight and BMI in these subjects (Katus *et al*., 2020a). It appears important to observe the dynamics of these relationships as the subjects age.

Earlier analysis on the same sample had revealed that Insatiability by Reward and both of its components were significantly positively associated with body weight and BMI (Katus *et al*., 2020a). This aspect of reward sensitivity did not have any similar associations with blood measures of carbohydrate or lipid metabolism as the other. A positive relationship between this fixation to reward measure was present specifically with TRG levels. It was also not related to habitual physical activity and was, not surprisingly given the association with BMI, negatively correlated with cardiovascular fitness. Nonetheless, Insationability by Reward was in inverse association with nutrient intake if this was adjusted to body weight. Hence, it could be assumed that this aspect of reward sensitivity is related to metabolic efficiency, this being lower in subjects with higher Insatiability. Openness to Rewards, in contrast, was not associated with nutrient intake; however, its facet with a more general nature had a trend for a positive association with protein intake, which was statistically significant for the proportional intake of protein in the diet. Disruption of the interaction between homeostatic and reward circuitry might promote overeating and contribute to obesity (Volkow *et al*., 2012), and obese individuals experience less activation of reward circuits from the actual food consumption, whereas they show greater activation of somatosensory cortical regions that process palatability when they anticipated consumption (Stice *et al*., 2008). The trait of fixation to a reward may subserve the association between ADHD and obesity (Chen *et al*., 2018), as far as specifically Insatiability by Reward was the reward sensitivity component that is associated with ADHD symptoms (Pulver *et al*., 2020a). Both facets could theoretically be associated with central dopaminergic neurotransmission, but their distinct nature is suggesting that variabilities in the genetically determined regulation of dopamine neurones (Gillies *et al*., 2014; Ghosal *et al*., 2019) underlie the independent variation in openness to multiple rewards and insatiability by a reward.

While it may be thought of as premature at this stage of investigation, it appears prudent to consider the public health implications of the findings. Previous research, from this laboratory among others, has suggested that efficacy of health behaviour interventions varies by individual differences in affective constructs (Paaver *et al*., 2013). Thus, consideration of components of reward sensitivity may help to tailor promotional projects for dietary behaviour or physical activity. However, much more work needs to be done, including the investigation of the potential interaction of reward sensitivity components with genetic or environmental factors (e.g. socio-economic status).

Conclusively, making a distinction between the two independent aspects of reward sensitivity, striving towards multiple rewards and fixation on particular rewards, can reveal the relationship of reward sensitivity with lifestyle, metabolic markers and possibly metabolism efficiency and may also help to explain why so many studies on implications of reward sensitivity have not yielded in consistent findings. The highly distinct association of the two aspects with a number of metabolically important variables indirectly suggests that these (sub-)traits are an integral part of the physiological homeostatic machinery of an individual and, by their independent variability within population, support variation in metabolism and lifestyle. Further studies should aim at understanding of whether and how are these associations causal, first gaining knowledge on the underlying biological mechanisms in order to guide the design of experimental models.
